# A Bayesian method for identifying associations between response variables and bacterial community composition

**DOI:** 10.1371/journal.pcbi.1010108

**Published:** 2022-07-06

**Authors:** Adrian Verster, Nicholas Petronella, Judy Green, Fernando Matias, Stephen P. J. Brooks

**Affiliations:** 1 Bureau of Food Surveillance and Science Integration, Food Directorate, Health Products and Food Branch, Health Canada, Ottawa, Canada; 2 Bureau of Nutritional Sciences, Food Directorate, Health Products and Food Branch, Health Canada, Ottawa, Canada; University of Virginia, UNITED STATES

## Abstract

Determining associations between intestinal bacteria and continuously measured physiological outcomes is important for understanding the bacteria-host relationship but is not straightforward since abundance data (compositional data) are not normally distributed. To address this issue, we developed a fully Bayesian linear regression model (BRACoD; Bayesian Regression Analysis of Compositional Data) with physiological measurements (continuous data) as a function of a matrix of relative bacterial abundances. Bacteria can be classified as operational taxonomic units or by taxonomy (genus, family, etc.). Bacteria associated with the physiological measurement were identified using a Bayesian variable selection method: Stochastic Search Variable Selection. The output is a list of inclusion probabilities ($\hat{p}$) and coefficients that indicate the strength of the association (${\hat{\beta }}_{included}$) for each bacterial taxa. Tests with simulated communities showed that adopting a cut point value of $\hat{p}$ ≥ 0.3 for identifying included bacteria optimized the true positive rate (TPR) while maintaining a false positive rate (FPR) of ≤ 5%. At this point, the chances of identifying non-contributing bacteria were low and all well-established contributors were included. Comparison with other methods showed that BRACoD (at $\hat{p}$ ≥ 0.3) had higher precision and a higher TPR than a commonly used center log transformed LASSO procedure (clr-LASSO) as well as higher TPR than an off-the-shelf Spike and Slab method after center log transformation (clr-SS). BRACoD was also less likely to include non-contributing bacteria that merely correlate with contributing bacteria. Analysis of a rat microbiome experiment identified 47 operational taxonomic units that contributed to fecal butyrate levels. Of these, 31 were positively and 16 negatively associated with butyrate. Consistent with their known role in butyrate metabolism, most of these fell within the Lachnospiraceae and Ruminococcaceae. We conclude that BRACoD provides a more precise and accurate method for determining bacteria associated with a continuous physiological outcome compared to clr-LASSO. It is more sensitive than a generalized clr-SS algorithm, although it has a higher FPR. Its ability to distinguish genuine contributors from correlated bacteria makes it better suited to discriminating bacteria that directly contribute to an outcome. The algorithm corrects for the distortions arising from compositional data making it appropriate for analysis of microbiome data.

## Author summary

We present a fully Bayesian linear regression model to identify associations between physiological measurements (continuous data) and intestinal bacteria (relative bacterial abundances; compositional data). The BRACoD (Bayesian Regression Analysis of Compositional Data) algorithm corrects for the compositional nature of the bacterial data to provide a list of inclusion probabilities and regression coefficients that indicate the strength of the association. If desired, the user can specify a cut point to select only the bacteria that meet predetermined performance characteristics. Analysis of a simulated dataset based on data from a rat microbiome study indicated that an inclusion probability cut point value ≥ 0.3 minimized the false positive rate while maintaining a reasonably high sensitivity (true positive rate). The identified associations form a starting point for generating hypotheses about the relationship between the gut microbial community and physiological outcomes.

## Introduction

Identifying bacterial taxa associated with physiological outcomes, such as the production of a metabolite, is important since these associations are the starting point for generating hypotheses about the relationship between the gut microbial community and physiological outcomes. There are many methodological issues [1] and interpretive assumptions [2] that must be considered when examining the relationship between relative abundance data (either 16S rRNA or metagenomics) and physiological outcomes. However, one important aspect is often neglected: bacterial analysis is compositional because the relative abundance of all bacteria sum to 100% [3]. This means that there are severe statistical distortions when common procedures are used to identify associations among bacteria or correlations with physiological outcomes [3]. While a number of methods have been suggested to overcome this problem when analysing associations among bacteria [4–6], methods that identify associations with metabolite levels or physiological outcomes have been lacking until recently [7, 8]. Although algorithms have been developed for correlation analysis of compositional data [9–11], these are not suitable when seeking to define associations with metabolic or physiological outcomes. Most problematic is the use of Pearson correlations to identify bacteria-metabolite correlations even after normalizing the data [12] because it generates high numbers of false positives. This makes it difficult to distinguish important relationships from background noise.

Here, we present a Bayesian regression algorithm that identifies bacteria associated with continuous physiological outcomes. Following the method of BAnOCC [6], the current algorithm transforms compositional data using an unobserved total abundance variable. The algorithm was tested using measured bacterial changes and fecal butyrate concentrations as a function of diet in rats. Short chain fatty acids, and in particular butyrate, are a focus of attention because they have been implicated in the development and maintenance of immunity [13, 14] and inflammation [15, 16]. It also appears that they play essential roles in physiological function [17]. Reflecting this, researchers have studied the effect of different dietary colonic fermentable material on bacterial community composition with the aim of increasing SCFA (especially butyrate) [17–20]. Interpretation of results is complex as several factors can influence the production of fermentation end products, including the rate of fermentation, the degree of oxidation of the substrate, and the amount of substrate [21]. In addition, while only specific bacterial families/species have the ability to produce butyrate [22–24], non-producing bacteria may act synergistically with butyrate producers to stimulate production. For example, butyrate producing bacteria have been shown to use lactate produced by some strains of bifidobacteria in co-culture [21, 25, 26] or to take advantage of other bacteria’s ability to digest longer-chain carbohydrates [25]. The combination of all these factors makes *in vivo* identification of bacteria associated with butyrate production difficult, highlighting the need for a robust statistical method for correctly assessing relationships. Whereas the present work uses butyrate as a response variable, the method can be applied to any system where a continuous variable is related to compositional measures. This could include fermentation end products or immune system parameters measured as a function of 16S rRNA abundance data.

### Design and implementation

#### Ethics statement

The animal protocol was approved by the Health Canada Animal Care committee.

#### Bayesian method

**Fig 1:**
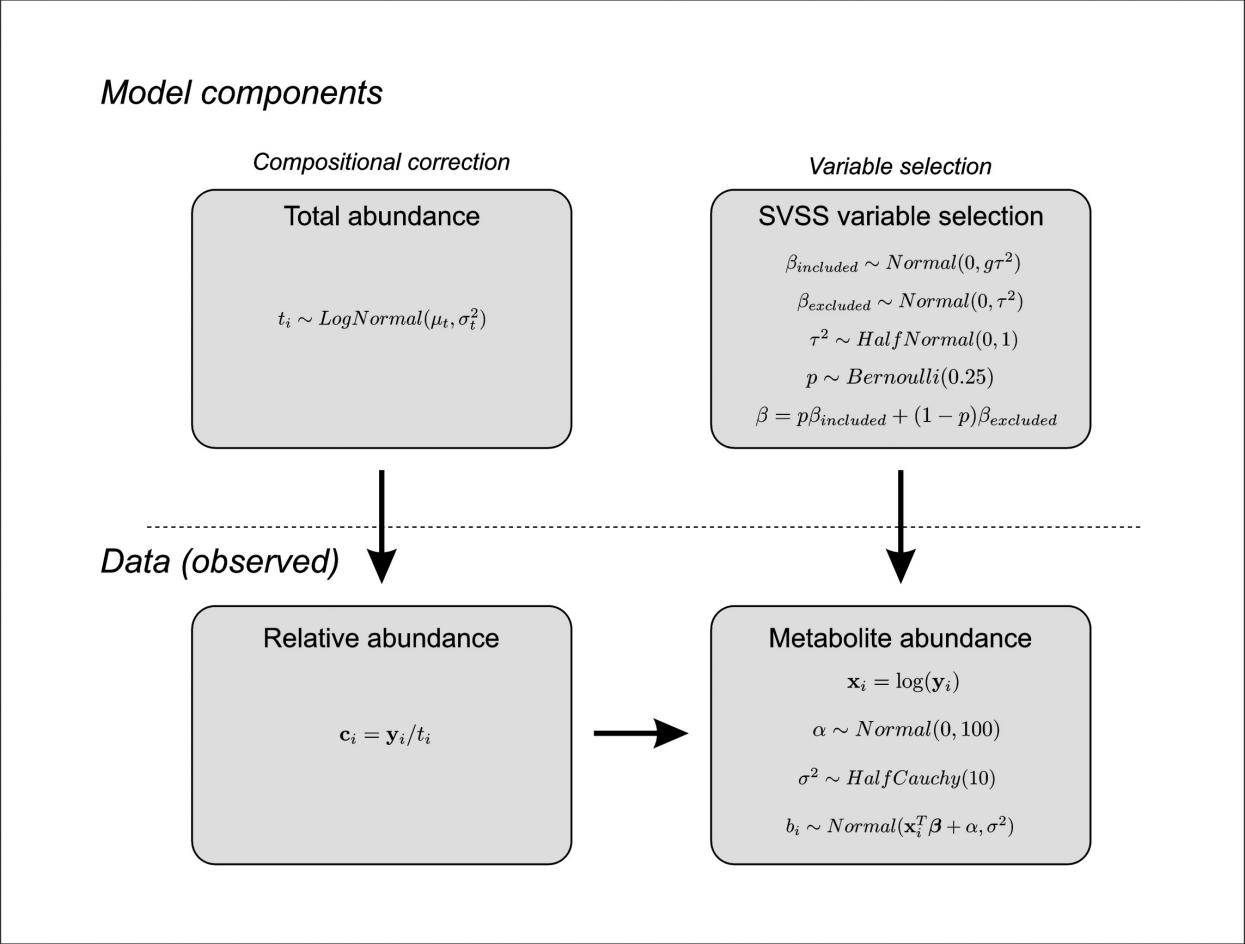
Outline of the BRACoD method to identify bacteria associated with a physiological outcome. Known data includes the relative abundances of bacterial groups at a defined level of classification (*c*_*i*_ for microbiome sample *i*) and sample-specific measurements of a physiological outcome (*x*_*i*_). The model uses several unobserved variables to identify associations between the two sources of data. First, to correct for the compositional nature of the data, relative abundances are related to absolute abundances by an unknown per sample total abundance, which is modelled with a lognormal distribution with hyperparameters μ_t_ and σ^2^_t_ (although, in the final form of the likelihood equation, this variable was marginalized out; see Methods). To identify the associations, the physiological outcome is modelled as linearly related to the log absolute abundance of each of the bacterial species via unobserved regression coefficients which are inferred by the model. Contributing bacteria are selected using a stochastic search variable selection (SSVS) procedure where the Bernoulli variable *p* designates if a given bacteria is a contributor or a non-contributor. The Bernoulli variable controls whether or not the regression coefficient is included (non-zero) or excluded (forced to zero).

A fully Bayesian regression model (BRACoD: Bayesian Regression Analysis of Compositional Data) was developed to model the relationship between physiological outcomes and relative bacterial taxa abundances across a number of environments (Fig 1). The program reports two important numbers that describe the association between bacteria and a physiological outcome: (1) a metric indicating whether an association exists; and (2) the strength of that association. Bacteria can be identified as operational taxonomic units (OTUs) or as a taxonomic classification (genus, family, etc.). The total abundance of each environment was modelled using a log normal distribution to more accurately reflect the distribution of OTUs in a typical sample.

Feature selection (selection of bacteria classifications associated with butyrate concentrations) was performed using a spike and slab approach called the Stochastic Search Variable Selection [27]. This approach is similar to LASSO, a widely used frequentist method that selects features using a penalty term that sets as many features as possible to zero [28]. However, instead of using a penalty term, the BRACoD algorithm identifies excluded bacteria by assigning them to a narrow, low variance distribution that is nearly zero (the spike), and identifies included bacteria by assigning them to a wide, high variance distribution (the slab) that models genuine contribution coefficients. Variables are selected based on their explicit attribution to one or the other distribution using a 0 or 1 assignment variable. In detail, the included and excluded coefficients are assigned as:

$$\beta_{excluded}∼Normal(0,\tau^{2})$$

$$\beta_{included}∼Normal(0,g\tau^{2})$$

where *τ*^2^~*HalfNormal*(0,1) and g is a large number (set to 500). To select between the included and excluded version of the regression coefficient for each bacterial classification, BRACoD uses a Bernoulli random variable that assigns either *β*_*included*_ or *β*_*excluded*_ to be used in the regression model:

p∼Bernoulli(0.25)

$$\beta =p\beta_{included}+(1-p)\beta_{excluded}$$ (1)

Pre-transformations are commonly used to account for the compositional nature of the data, but following Schwager et al. [6], BRACoD instead uses an unobserved absolute abundance variable. Furthermore, similar to Schwager et al. [6], BRACoD marginalizes the total environment abundance variable to speed up the Markov chain Monte Carlo (MCMC) sampling (see Fig 1 and S1 File). This results in a likelihood function:

$$L=1/\sqrt{2\pi \sigma^{2}}\times exp\left\{-\frac{1}{2}(-\sum_{i}\frac{b_{i}^{2}}{a_{i}}+{\left\|y-\alpha -log(C)\beta \right\|}^{2})\right\}\prod_{i}\sqrt{\frac{2\pi }{a_{i}}}$$ (2)

$$a_{i}=\frac{\sum_{j}w_{j}}{\sigma^{2}}+\frac{1}{\sigma_{t}^{2}}$$

$$b_{i}=\frac{\left(\sum_{j}\beta_{j})(y_{i}-\alpha -{\log\left(c_{i}\right)}^{T}\beta \right)}{\sigma^{2}}+\frac{\mu_{t}}{\sigma_{t}^{2}}$$

where y is the vector of butyrate metabolites, ***C*** is a matrix of bacterial relative abundances (across sample *i* and bacteria *j*), ***β*** is a vector of regression coefficients (*β*), and *μ*_*t*_ and $\sigma_{t}^{2}$ are the prior parameters of the unknown total abundance.

Instead of identifying a single maximum likelihood estimate as frequentist models do, Bayesian models infer the value of model variables by characterizing by the distribution of the posterior of each random variable. PyMC3 [29] was used to implement BRACoD and sample the posterior, which draws MCMC samples using the No-U-Turn Sampler on continuous variables [30] and the Binary Metropolised Gibbs Sampler [31] on the binary variable ***p*** (1 or 0, depending on whether the variable was included). To identify elements of ***C*** that were associated with the response vector ***Y***, the binary inclusion parameter *p* was averaged across posterior samples to determine an inclusion probability ($\hat{p}$) that ranges from 0 to 1. The regression coefficient of the included bacteria (${\hat{\beta }}_{included}$) was averaged over the posterior distribution of *β*_*included*_ to obtain a value for the strength of the relationship.

#### Rat feeding trial

A total of 175 male Sprague-Dawley rats (Charles River, Saint Constant, QC) were fed for 2 weeks on a high fat diet and then fed for 10 weeks with one of 5 diets (wood cellulose, hard red spring wheat bran, oat bran, high amylose maize starch or short chain fructooligosaccharides) calculated to give 7% of total dietary fibre in each diet (Dyets, Inc.; Bethlehem PA; Table A in S1 File). Animals had free access to food and water. After the feeding trial, animals were sacrificed by exsanguination while under anesthesia and their fecal contents removed and frozen in liquid nitrogen. Samples were stored at -80°C until analysis for short chain fatty acid (SCFA) profiles by HPLC [32]. Fecal (distal colon) samples were ground in liquid nitrogen [33] and community DNA was isolated using the QIAgen faecal DNA isolation kit carried out according to the manufacturer’s procedure for difficult to lyse bacteria (QIAgen, Toronto, ON). Isolated community DNA was quantified using PicoGreen (Fisher Scientific Co, Ottawa ON) and stored frozen at -20°C. Purified DNA was subjected to 16S rRNA gene analysis (MiSeq) using standard protocols as recommended by the manufacturer (Illumina). The 16S rRNA V4 region was amplified using primers and conditions as previously described [34]. Amplicons were bi-directionally sequenced, quality filtered, assembled, and the primer sequences removed (~240 bp reads). Raw sequences were processed [35] and aligned to existing taxa within the Silva database (MOTHUR; [1]).

#### Simulations

BRACoD accuracy was evaluated by simulating the relative abundances of microorganisms, some of which contribute to butyrate production and by simulating the bacteria’s ability to produce an environmental variable (i.e., butyrate in this example). Two commonly used distributions were combined to simulate microbial abundances in this study: lognormal distribution [36] and Dirichlet-multinomial distribution [37]. The method was as follows. First, for each sample (i), a log transformed absolute bacterial abundance distribution was simulated using a normal distribution

$$y_{i}=lnx_{i}∼Normal(\mu ,\Sigma )$$ (3)

The mean and variance parameters were empirically derived from a log transformed version of the dataset at hand so that the absolute abundances will follow a lognormal distribution. In this way, the simulated dataset replicated experimentally measured rat microbiome data. In the case when correlations between bacteria were not included, the covariance matrix is diagonal. When the model included a correlation between the abundance of bacteria j and bacteria k, off-diagonal elements of the covariance matrix were included according to the definition of correlation

$${\sigma^{2}}_{jk}=\rho_{jk}*\sigma_{j}*\sigma_{k}$$ (4)

The simulation was restricted to bacteria whose average relative abundance in the dataset was greater than approximately 0.1% to exclude minor bacterial components and speed up the test calculations. Second, the absolute abundance values were used to generate levels of butyrate based on a linear equation.


$$b_{i}∼Normal(\beta^{T}x,\tau^{2})$$

$$\beta_{j}∼Uniform(-5,5)\;For\;included\;bacteria$$

$$\beta_{j}=0\;For\;excluded\;bacteria$$

Bacteria that did not contribute to butyrate production had a coefficient of zero, while contributing bacteria had their coefficients drawn from a uniform distribution between -5 and 5. Third, the simulated absolute bacteria abundances were normalized to relative abundances and read counts for each bacteria were simulated using a multinomial distribution.


$$c_{i}=\frac{y_{i}}{\sum y_{i}}$$ (5)

$$counts_{i}∼Multinomial(c_{i},n_{reads})$$

It should be noted that the simulated community distributions are dependent on the microbiome dataset used as the basis for the process.

Using simulated data enabled an assessment of BRACoD’s performance across several parameters because it allowed direct control of variables related to the production of butyrate, including: the number of butyrate-producing OTUs, the relative activity of the OTUs, the number of rats sampled in an experiment, and the sequencing depth. The relationship between producing and non-producing OTUs was controlled through the strength of the inter-bacterial correlations. The simulated data had a similar distribution of detected (non-zero count) OTUs, average abundance, and coefficient of variation (Fig A in S1 File).

#### Algorithm performance and comparison with other methods

In order to evaluate the ability of BRACoD to distinguish between contributing and non-contributing bacteria and to evaluate its relative performance, BRACoD results were compared to normalized data (centered log ratio transformed; [3]) using LASSO for variable selection [28] (clr-LASSO) and a linear model with a spike and slab variable selection algorithm from the ’BoomSpikeSlab’ package in R after centred log transformation (clr-SS). LASSO is commonly used for variable selection in the microbiome field but spike and slab methods are rarely used, and we include it as a comparison because it forms a closer comparison to the model we’ve constructed. In the clr-SS model, contributing bacteria were identified as those with an inclusion probability above 0.3, which is the same cut point used in BRACoD. All simulations were run with 1000 iterations. The tests included: varying the number of bacteria that contribute to the environmental outcome, changing the strength of the relationship between a bacterial taxon and the environmental outcome (by changing the simulated regression coefficient), increasing the number of bacterial taxa in the community (by lowering the abundance threshold to include less abundant taxa in the community), and examining the influence of bacteria that are correlated with contributors but are not contributors themselves. Method parameters were calculated as follows:

TPR(TruePositiveRate)=TP/P (6)

where TP = the number of correctly identified positives and P = the number of real positive cases

FPR(FalsePositiveRate)=FP/N (7)

where FP = the number of falsely identified positives and N = the number of real negative cases

precision=TP/(TP+FP) (8)

accuracy=(TP+N‐FP)/(P+N) (9)

## Results

### Simulation tests

**Fig 2:**
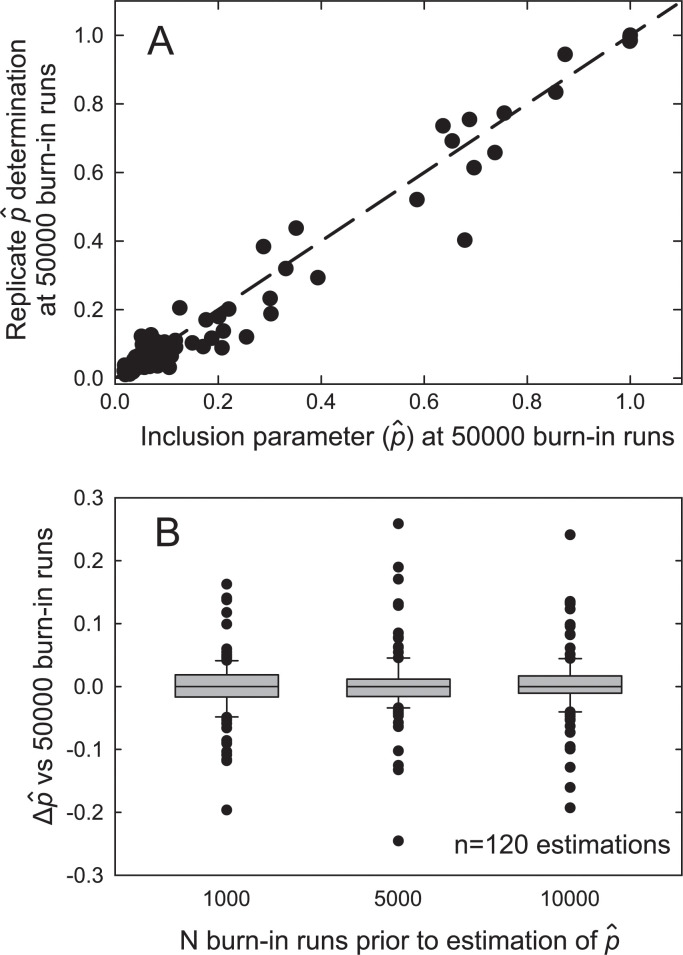
Reproducibility and effect of burn-in on BRACoD performance. Fig 2A: The inclusion probabilities from two separate BRACoD runs using the same set of simulated data are plotted against each other. Regression analysis showed good agreement between the runs: y = 0.994 × $\hat{p}$ (first run)– 0.0083; adjusted r^2^ = 0.981. Fig 2B: Box and whisker plot showing difference in $\hat{p}$ values for various numbers of burn-in runs vs 50000 burn-in runs as reference. Values for 1000, 5000, and 10000 burn-in runs were not significantly different from that for 50000 burn-in runs.

Simulated communities were constructed using relative OTU data and butyrate concentrations obtained from a rat feeding trial (see Table A in S1 File for diets and S2 and S3 Files for data). The predictors were the fractional abundances of the OTUs from each rat and the response variable was the corresponding fecal butyrate concentration. The parameters of interest were the distribution of the inclusion probability ($\hat{p}$) and the regression coefficient of the included bacteria (${\hat{\beta }}_{included}$). The first test addressed the algorithm’s convergence and reproducibility. This was necessary because Bayesian algorithms are not deterministic. The stability and reproducibility of the program output was demonstrated by a high correlation among inclusion parameter values from replicate runs of the same data ($\hat{p}$; Fig 2A). This reproducibility implied that the MCMC did not suffer from an overly complex posterior. It was also important to determine the length of the required burn-in period before the posterior can be accurately characterized. Tests showed that the method performed well with a 1000 burn-in steps (Fig 2B).

### Cut point selection

**Fig 3:**
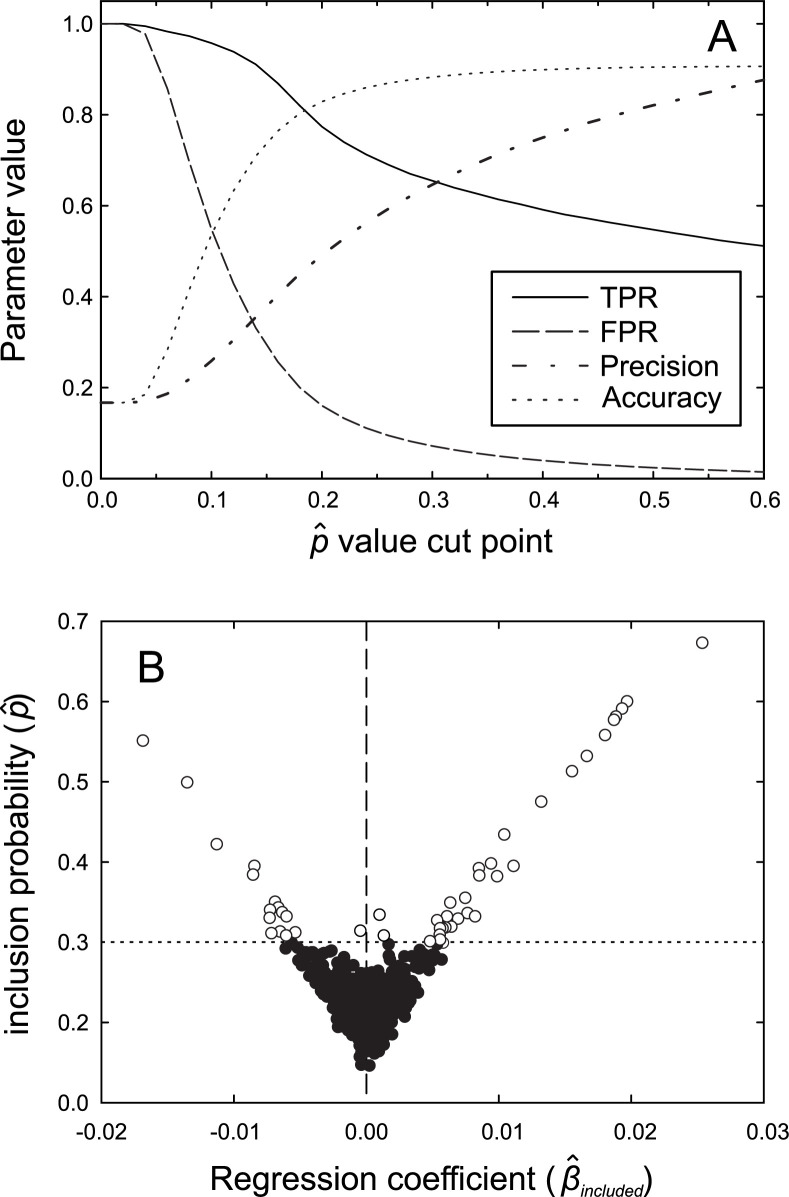
Cut point estimation. Fig 3A: Method performance measures are plotted as a function of the $\hat{p}$ cut point value. Data is from 1000 microbiome datasets simulated for a community of N = 20 contributing bacteria and N = 100 non-contributors. Fig 3B: Plot of the inclusion probability ($\hat{p}$) as a function of the regression coefficient (${\hat{\beta }}_{included}$) obtained from a BRACoD analysis of rats fed diets differing in dietary fiber. Visual inspection of the distribution of ${\hat{\beta }}_{included}$ values around the origin (vertical dashed line) suggested a ${\hat{\beta }}_{included}$ cut point ≈ 0.006 corresponding to a $\hat{p}$ value cut point of 0.3 (dotted horizontal line). Contributors (points falling above this value) are represented as open circles while non-contributing OTUs (below cut point) are represented as filled circles.

A cut point value for $\hat{p}$ can be useful in distinguishing associated from non-associated bacteria because the inclusion parameter ($\hat{p}$) is continuous rather than binary. This empirical cut point will reflect individual preferences since it is set by the user. Ideally, it represents a balance of overall performance metrics, including, but not limited to, the TPR, the FPR, and the correlation between bacteria and the physiological outcome. In our analysis, the cut point was chosen based on the highest TPR that corresponded to a low FPR. At this point, one should have high confidence that the bacteria identified by the algorithm are actually associated with the outcome. This point should also exclude bacteria not related to the physiological outcome (low FPR). Simulations showed a broad performance optimum starting around 0.3 with a high accuracy, and low FPR and a slow decline in the TPR (Fig 3A). Note that the user must choose the balance among method performance characteristics. We tend to prefer a low FPR while maximizing the TPR and accuracy. A visualization that was helpful in deciding on the cut point value was a plot of $\hat{p}$ vs ${\hat{\beta }}_{included}$ obtained from BRACoD. Plots were generated for each short chain fatty acid: butyrate (Fig 3B) and acetate, propionate and isobutyrate (Fig B in S1 File). It was apparent that the tight clustering of points in the region -0.006 < ${\hat{\beta }}_{included}$ < 0.006, corresponding to a value of $\hat{p}$ < 0.3, made it difficult to distinguish associated from non-associated bacteria but data outside this range were relatively well separated. This point was associated with a TPR/FPR ratio ≈ 7 and low FPR (<5%) in simulated communities lending confidence to its choice for correctly identifying associated bacteria in this community. By definition, at $\hat{p}$ = 0.3 an OTU was included in 30% of the chains, suggesting it provides a reasonable value for discriminating associated from non-associated bacteria.

### Algorithm performance and comparison with other methods

**Fig 4:**
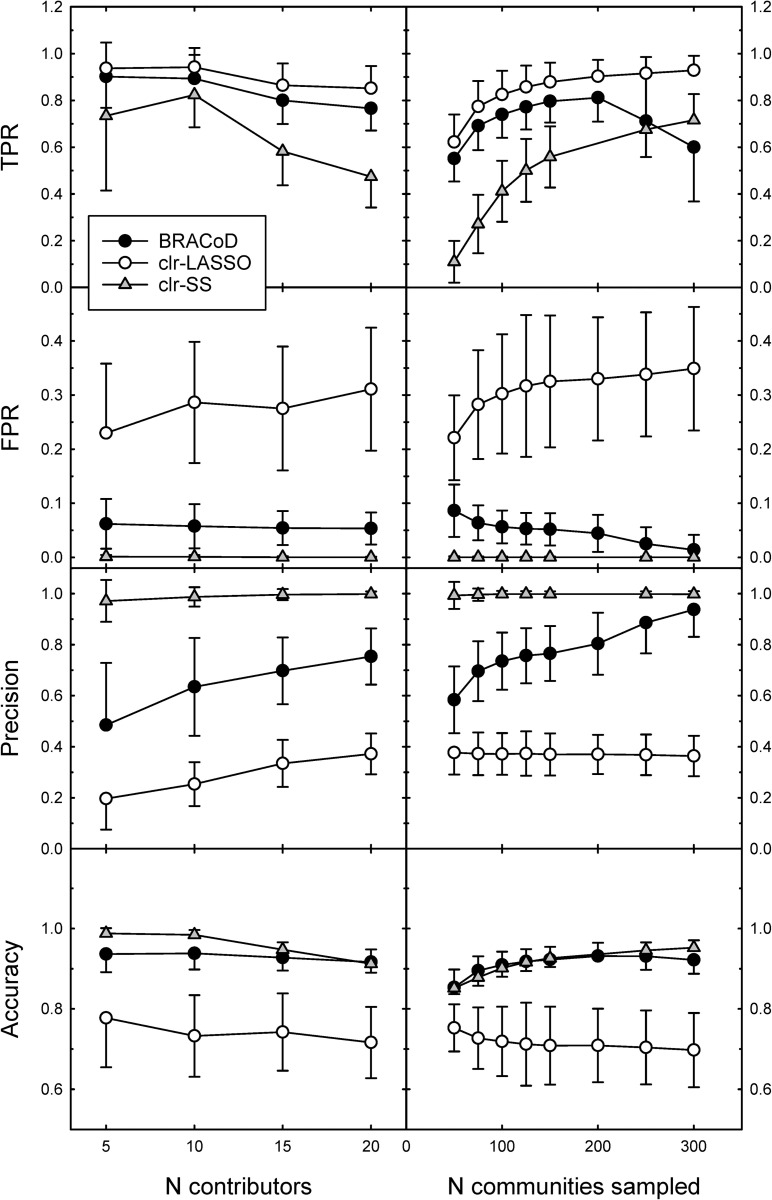
Parameter metrics in simulated communities. Method performance measures are plotted as a function of the number of contributing bacteria (left panels) or the total number of bacterial communities sampled (right panels). Values represent means ± SD for 1000 simulations with 100 non-contributing bacteria. Inclusion cut point for BRACoD = 0. For left hand panels, the number of community samples was set to 119, the number of rats in the bacterial dataset.

Independent of the number of pre-determined contributors, BRACoD had a higher TPR, a lower FPR, and higher precision and accuracy than the clr-LASSO method (Fig 4, left panels). The lower precision of clr-LASSO was due to incorrect classification of non-contributing taxa (higher FPR and lower TPR). On the other hand, BRACoD had a higher TPR than the clr-SS method but a higher FPR and lower precision. The higher precision of clr-SS was due to its low FPR which was achieved at the expense of excluding all but the most highly correlated bacteria. These characteristics are reflected in a higher ROC-AUC for BRACoD compared to the other methods: 0.9114 (BRACoD) vs 0.8798 (clr-LASSO) and 0.8162 (clr-SS; Fig C in S1 File).

Varying the strength of the relationship between contributing bacterial taxa and the environmental outcome showed a negligible effect on BRACoD performance. Increasing the total number of communities sampled (essentially increasing the total number of subjects in the experiment) showed a fairly stable optimum between 100 and 200 samples (Fig 4 right panels). It should be noted, however, that (as expected) the algorithm’s run time increased significantly with increasing numbers of community samples. We noted that clr-SS had exceptionally poor TPR values when the number of community samples was below 100, a situation likely to occur in many experimental designs. Both FPR and Precision improve with increasing numbers of samples for BRACoD but remain virtually constant for the other algorithms.

**Fig 5:**
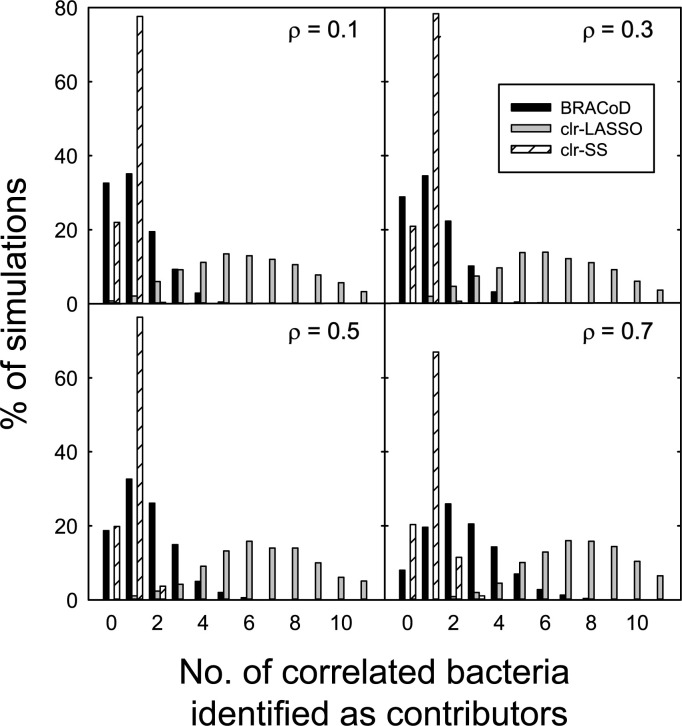
Ability of the algorithms to distinguish correlated bacteria from true contributors. The percentage of simulations (N = 1000) that identified non-contributing correlated bacteria as contributors is shown for correlation strengths between 0.1 and 0.7 (ρ in the equation: ${\sigma^{2}}_{jk}=\rho_{jk}*\sigma_{j}*\sigma_{k}$; see Simulations section). Contributing bacteria were identified as having $\hat{p}$ ≥ 0.3. Higher values to the left of each panel indicate a superior ability to discriminate between contributors and non-contributors. Simulated data were constructed with 20 contributors plus 100 non-contributing bacteria. Within the non-contributing bacteria, 20 were chosen as correlated bacteria.

A common problem when looking for statistical associations involves distinguishing between correlation and causation. In bacterial-outcome analyses, this could arise if bacteria that correlate with outcome-associated bacteria are wrongly identified as associated bacteria. For example, in the case of butyrate production, it is possible that non-producing bacteria that correlate with butyrate producing bacteria are identified as producers. This would increase the FPR and lower precision. The magnitude of this effect was assessed using data constructed with identified contributing bacteria and correlated (non-producing bacteria). The BRACoD algorithm detected few of the correlated bacteria even at relatively high correlation strength (Fig 5) showing that BRACoD exhibits a good balance between TPR and precision compared to alterative methods. This phenomenon was further investigated to determine the characteristics that distinguish BRACoD-identified from non-identified bacteria. The results showed that BRACoD is much more likely to successfully identify bacteria that make a large contribution to butyrate production (Fig D in S1 File). This makes intuitive sense; it should be easier to identify bacteria that produce or consume large amounts of butyrate compared of those that only produce or consume a small amount.

### Application to microbiome data in rodents

A total of 119 fecal samples were sequenced from rats fed diets differing in fat and dietary fibre (Table A in S1 File) generating 18.8 million sequences that resolved into 469 OTUs. Acetate, propionate, butyrate, and isobutyrate concentrations per gram dry weight feces were used as physiological outcomes. Using this dataset 47 OTUs ($\hat{p}$ ≥ 0.3; Table B in S1 File) were identified. For butyrate, a strong relationship existed between the probability of inclusion in the posterior ($\hat{p}$) and the mean regression coefficient of the included bacteria (${\hat{\beta }}_{included}$; Fig 3B) showing that, similar to the case with simulated data, strongly associated bacteria are more likely to be included in the converged solution. The majority of identified OTUs fell within the Lachnospiraceae and Ruminococcaceae families, although most were detected at low abundance: only 8 OTUs had an abundance > 0.1% (Table B in S1 File). The identification of similar bacterial species across the diets is interesting and suggests that the same bacteria contribute to butyrate production regardless of the source of dietary fiber.

Similar results were obtained when analysing for bacterial species related to acetate, propionate and isobutyrate, however the ${\hat{\beta }}_{included}$ coefficients showed a SCFA-specific pattern (Fig B in S1 File). For example, there was a clear separation between associated and non-associated bacteria at the 0.3 cut point for acetate and the maximal $\hat{p}$ values were higher suggesting a stronger association. Similar trends were observed for propionate but there was a less apparent distinction at the 0.3 cut point. In this latter case, a bacterium with relatively high $\hat{p}$ values (>0.7) with and ${\hat{\beta }}_{included}$ coefficient of -0.03 suggested that an OTU within the Lachnospiraceae family plays a relative large role in propionate disappearance. Analysis of bacteria related to isobutyrate showed relatively lower inclusion probabilities (56 OTUs; 0.3 ≤ $\hat{p}$ ≤ 0.54) with ${\hat{\beta }}_{included}$ coefficients between -0.017–0.012. This suggests a relatively equal role in isobutyrate metabolism.

### Availability and future directions

BRACoD and the code for simulating bacterial community abundance (with corresponding butyrate levels) is available with interfaces in both R and Python on GitHub at https://github.com/ajverster/BRACoD.

### BRACoD performance

Identifying bacteria that are associated with a physiological outcome is an important step in hypothesis generation. For bacterial community distribution data, finding these relationships is complicated due to the compositional nature of the data. Normalization is possible using transformations such as the centered log ratio [3, 38] but this seems to falsely identify contributors in many instances, giving an artificially high number of significant correlations. A recently published robust regression procedure does not suffer this difficulty [7, 8] but a limited analysis with our simulated data gave 40% precision and 35% TPR (compare values of around 65% at a cut point = 0.3 in Fig 3A). In this paper, we describe a Bayesian approach that performed well with simulated communities. The algorithm identifies both the likelihood of an association as well as the uncertainty of the contribution coefficient by characterizing the posterior distribution of the model using MCMC. To identify whether a bacterial taxon is a contributor to a response, the model uses a form of a Bayesian feature selection method known as spike and slab: the Stochastic Search Variable Selection (SSVS). Spike and slab methods work by modelling two populations of regression coefficients, a distribution tightly surrounding zero of excluded coefficients (the spike) and a broad distribution for the included variables (the slab). Bacteria are assigned to one of these two populations using a selection variable, which explicitly determines which bacteria are associated with the dependent variable and which are not. This is a fundamentally different approach from LASSO based methods which identify associated bacteria by assigning the coefficient of non-associated bacteria to zero. Similar to previous work [6], we corrected for the compositional problem by modelling an unobserved total abundance variable. This is also different than the clr-SS approach, which requires data transformation because the algorithm cannot be directly applied to compositional data. Using MCMC to characterize the posterior distribution allows the model to identify bacteria which associate with higher butyrate concentrations since these bacteria have higher inclusion probabilities ($\hat{p}$).

Simulations can inform experimental design because they indicate the number of samples and the degree of sequencing depth required for performance of the model. This is intuitively similar to a power calculation, although not identical because Bayesian approaches do not identify positives by rejecting the null hypothesis. Using simulated data, we observed that BRACoD sensitivity (TPR) was approximately constant between 100 and 200 bacterial communities sampled. Over this range, the FPR was around 5%, precision varied between 70–80%, and accuracy was approximately 90% (Fig 4, right panels). This appears optimal since we believe that a cut point with a low FPR and a high TPR will favour identifying the most strongly associated bacteria while avoiding bacteria that have only a weak association with the environmental variable. These conclusions were drawn from analyses using a cut point of 0.3, which was optimal for this data set but may vary with different experimental set ups (differing numbers of contributors and communities sampled).

An advantage of the BRACoD algorithm is that it allows the user to choose a preferred balance between sensitivity (TPR) and FPR. This is a significant improvement over the clr-SS algorithm which is largely refractive to the inclusion parameter value (Fig E in S1 File), and to clr-LASSO where the inclusion parameter is determined automatically using the cross validation function. As mentioned above, it is possible that a cut point of 0.3 is not optimal for all experimental designs. The effect of the number of included bacteria and total number of community samples on method performance parameters (Fig 4) may mean that the cut point should be optimized to reflect the individual experimental parameters. This can be accomplished by setting the ‘cutoff’ value to 0.0 in the summarize_trace function. This will produce a list all the bacteria with their associated $\hat{p}$ and ${\hat{\beta }}_{included}$ values. Construction of plots similar to Fig 3B will help confirm the selection of the cut point value (if one is desired). It should be noted that the most highly associated bacteria will almost always be identified as contributors while those with lower $\hat{p}$ values will not always be included in the final list. It is important to remember this when interpreting the algorithm’s output. The cut point of 0.3 does not represent a defining line between contributors and non-contributors but was chosen to maximize BRACoD’s performance using a known community. Thus, the taxa with $\hat{p}$ > 0.3 represent taxa of interest for further experimentation rather than taxa known to affect an outcome.

It should be noted that BRACoD precision was highest when both positive and negative contributors were included as opposed to the situation when only positive or only negative contributors were included in the simulation. This is similar to frequentist methods that identify positively and negatively correlated bacteria. It is apparent that the BRACoD algorithm balances the effect of the positive contributors by assigning negative contributors during the fitting process. Thus, the algorithm’s apparent requirement to include positive and negative contributors means that it will always identify (positive or negative) associated bacteria to balance the calculation even if they are not in the dataset. This is a consequence of the way in which the algorithm works: BRACoD assigns positive and negative contributors based on their relationship to the outcome. Thus, bacteria associated with lower butyrate levels are assigned a negative value while bacteria associated with higher butyrate levels are assigned a positive value. While this phenomenon has not been described in the literature on butyrate producing bacteria, this probably reflects actual microbial community dynamics. Further investigation, which could involve *ex vivo* fermentation reactions with isolated bacteria, are required to confirm these results. Several reasons for a negatively associated bacteria are possible. For example, a negative coefficient may mean that a bacterium effectively out-competes a butyrate producing bacteria for ecological space within the community or it may mean that the bacterium directly consumes the outcome product (e.g., butyrate in this example) or a substrate required for its production. While it is most likely that both butyrate producers and butyrate consumers exist in the gut microbiome, researchers should be mindful of this aspect of the analysis and treat butyrate producers and consumers with some caution. This should be kept in mind especially if a different (lower) cut point value is selected, since many more bacteria (that may or may not be associated with butyrate) will potentially be included.

### Interpreting output

A dataset from a rat feeding trial was analyzed to demonstrate the application of BRACoD to actual microbiome datasets. For this analysis, the full dataset of 469 OTUs were used. Using a cut point of 0.3, approximately 47 OTUs were identified as being associated with butyrate metabolism (Table B in S1 File). Although the properties of most of the OTUs could not be determined because they aligned with uncultured bacterial sequences, a strong correlation between OTUs falling within the Lachnospiraceae and Ruminococcaceae families and butyrate levels agrees with data showing that these families contain bacteria that directly produce butyrate [22, 24, 39] and are associated with fermentation of longer chain carbohydrate fibres. Many of these bacteria were present at low abundance suggesting that an analysis at the genus or family level may be more appropriate when identifying taxa important to SCFA production since bacteria at these classifications are expected to share many characteristics and functionalities. Performing the analysis at the genus level identified 11genera with $\hat{p}$ > 0.3 (Fig F in S1 File), which included unclassified Lachnospiraceae as the most abundant taxa (14.4% of total bacteria), followed by uncultured Lachnospiraceae (1.3%) and Ruminococcaceae (0.3%). The same analysis at the family level also showed a dominance of Lachnospiraceae (21% of all bacteria) along with small amounts of other families (adding up to of <1% of total bacteria). One should be careful when performing higher-level taxon analyses because this will combine potential positively and negatively correlated OTUs, although it is more likely that bacteria within a genus will have similar metabolic characteristics and substrate/product profiles. Identification of several bacterial OTUs associated with acetate and isobutyrate production was expected as it appears that several different taxa are involved in its production and metabolism, with a wide variation in the content of these groups among the rat experimental population [39, 40]. These types of analyses illustrate the value of identifying associated bacterial taxa in interpreting relationships among a complex community.

It should be noted that interpretation of the list of associated bacteria is not straightforward and depends on the physiological outcome. This is highlighted by considering short chain fatty acid production versus immunological outcomes. For short chain fatty acid production, it is expected that the included bacteria will be of sufficient abundance to account for the increased production. For this reason, the relative abundance of the included OTUs were in included along with their inclusion probabilities to aid in the interpretation of the butyrate associations (Table B in S1 File). On the other hand, it is unclear whether abundance is important in interpreting a different response such as presence of a specific antibody that may depend only on the presence or absence of a specific bacterial taxon. It may also be that the relative abundance at locations proximal to the cecum are more relevant. For example, the terminal jejunum and ileum have many Peyer’s patches that may facilitate interactions with the bacteria community at that location. Other factors must also be taken into consideration when interpreting BRACoD output, including: the relationship between relative species abundance and measured relative 16S rRNA abundance, the difference between actual abundance and relative bacterial abundance (potentially affected by changes in total bacterial load), and the problem of distinguishing between live and dead bacteria when measuring 16S rRNA relative abundance.

## Supporting information

S1 FileFile containing the derivation of equation 2 and supplemental tables and figures.**Table A.** Diet compositions for the animal phase. **Table B.** Bacteria associated with fecal butyrate excretion in rats. **Fig A** Characteristics of original rat community data and data simulated following the procedure described in the text. The average abundance of each OTU is determined from ~100 samples (or simulations). **Fig B** BRACoD results for acetate (top), propionate (middle) and isobutryate (bottom). The inclusion probability $\hat{p}$ is plotted as a function of the regression coefficient ${\hat{\beta }}_{included}$ determined from BRACoD analysis of rat experimental data. Dotted horizontal line shows cut point = 0.3. Contributors are represented as filled circles (black) while non-contributing OTUs are represented as open circles (white) using a cut point value of 0.3. **Fig C** ROC curves showing model performance on a single simulated dataset with 20 contributing bacteria and 119 samples. For BRACoD and clr-SS, the inclusion probability $\hat{p}$ was varied to obtain different TPR and FPR values. For clr-LASSO, the regularization strength (lambda) was varied. The points on the curve correspond to the cut points used to determine TPR and FPR. For BRACoD and clr-SS this was an inclusion probability ($\hat{p}$) > 0.3 and for clr-LASSO this was the best lambda identified by cross validation. Different versions of the simulated data produce different ROC curves (see Fig 4, which demonstrates performance metric variation across simulations). This figure was, therefore, generated using 100 simulations. The areas under the curve are: 0.9114 (BRACoD), 0.8798 (clr-LASSO) and 0.8162 (clr-SS). **Fig D** Relationship between the contribution coefficient and the percentage of contributing bacteria that were identified as contributors (dotted line). The percentage of contributing bacteria that were not identified as contributors is also shown (solid line). **Fig E** Method parameters as a function of inclusion cut point ($\hat{p}$) for BRACoD (top) and clr-SS (bottom) algorithms. BRACoD TPR, FPR, precision, and accuracy parameters (top) are from Fig 3A and are included for comparison. **Fig F** BRACoD analysis of the association between bacterial genera (top) and bacterial family (bottom). The inclusion probability $\hat{p}$ is plotted as a function of the regression coefficient ${\hat{\beta }}_{included}$ determined from BRACoD analysis of rat experimental data. Dashed horizontal line shows cut point = 0.3. Contributors are represented as filled circles (black) while non-contributing OTUs are represented as open circles (white) using the cut point value of 0.3.(DOCX)

S2 File OTU counts for each fecal community from the rat feeding trial. (CSV)

S3 File Fractional butyrate concentration (Butyrate/total SCFA) in fecal samples from the rat feeding trial. (CSV)

S4 File Data for the individual points for each figure is found under the appropriate tabs. (XLSX)
