# Supplementary material for: A Bayesian method for identifying associations between response variables and bacterial community composition
Source: PLoS Comput Biol. 2022 Jul 6;18(7):e1010108. doi: 10.1371/journal.pcbi.1010108 (PMC9307184; doi:10.1371/journal.pcbi.1010108)
Supplement: S1 File — Table A. Diet compositions for the animal phase. Table B. Bacteria associated with fecal butyrate excretion in rats. Fig A Characteristics of original rat community data and data simulated following the procedure described in the text. The average abundance of each OTU is determined from ~100 samples (or simulations). Fig B BRACoD results for acetate (top), propionate (middle) and isobutryate (bottom). The inclusion probability p^ is plotted as a function of the regression coefficient β^included determined from BRACoD analysis of rat experimental data. Dotted horizontal line shows cut point = 0.3. Contributors are represented as filled circles (black) while non-contributing OTUs are represented as open circles (white) using a cut point value of 0.3. Fig C ROC curves showing model performance on a single simulated dataset with 20 contributing bacteria and 119 samples. For BRACoD and clr-SS, the inclusion probability p^ was varied to obtain different TPR and FPR values. For clr-LASSO, the regularization strength (lambda) was varied. The points on the curve correspond to the cut points used to determine TPR and FPR. For BRACoD and clr-SS this was an inclusion probability (p^) > 0.3 and for clr-LASSO this was the best lambda identified by cross validation. Different versions of the simulated data produce different ROC curves (see Fig 4, which demonstrates performance metric variation across simulations). This figure was, therefore, generated using 100 simulations. The areas under the curve are: 0.9114 (BRACoD), 0.8798 (clr-LASSO) and 0.8162 (clr-SS). Fig D Relationship between the contribution coefficient and the percentage of contributing bacteria that were identified as contributors (dotted line). The percentage of contributing bacteria that were not identified as contributors is also shown (solid line). Fig E Method parameters as a function of inclusion cut point (p^) for BRACoD (top) and clr-SS (bottom) algorithms. BRACoD TPR, FPR, precision, and accuracy parameters [file pcbi.1010108.s001.docx]

**Mathematical derivations**

Assuming the regression follows

$\hat{y}= \alpha+X\beta$ (S1)

the parts of the posterior that contain t are the likelihood

$p(\mathbf{y}\mathbf{|}\sigma^{\mathbf{2}}\mathbf{,}\alpha,\boldsymbol{\beta}, \mathbf{t}\mathbf{)=}\frac{\mathbf{1}}{\sqrt{2\pi\sigma_{t}^{2}}}exp\left( \frac{-1}{2\sigma^{2}}\left\| \mathbf{y}-\alpha-\boldsymbol{X\beta} \right\|^{2} \right)$ (S2)

and the prior for t, which follows a log normal distribution

$p\left( \mathbf{t} \right)=\prod_{i=1} \frac{1}{t_{i}\sqrt{2\pi\sigma_{t}^{2}}}\exp\left( \frac{-1}{2\sigma_{t}^{2}}{(\log\left( t_{i} \right)-\mu_{t})}^{2} \right)$ (S3)

The goal is to expand these terms and complete the square in log(t) so that it can be marginalized and ignored in the final equation. We assume the transformed absolute abundances for a single sample are
**x_i_** = log(*t_i_*c_i_). We can then expand to give

$\hat{y}_{i}=\alpha+{\mathbf{x}_{\mathbf{i}}}^{\text{T}}\boldsymbol{\beta}=\alpha+{\log\left( \mathbf{c}_{\mathbf{i}} \right)}^{\text{T}}\boldsymbol{\beta}+log(t_{i})\left( \sum_{j} \beta_{j} \right)$ (S4)

Expanding the exponent of the likelihood gives

$$\sum_{i=1} {(y_{i}-\alpha-{\mathbf{x}_{\mathbf{i}}}^{\text{T}}\boldsymbol{\beta})}^{2}=\sum_{i=1} {(y_{i}-\alpha-{\log\left( \mathbf{c}_{\mathbf{i}} \right)}^{\text{T}}\beta-\log\left( t_{i} \right)\mathbf{1}^{\text{T}}\boldsymbol{\beta})}^{2}$$

$$=\left\| \mathbf{y}- \alpha\mathbf{1}-log(\mathbf{C})\boldsymbol{\beta} \right\|^{2}+\sum_{i=1} \left[ {log(t_{i})}^{2}\left( \sum_{j} \beta_{j} \right)-2log(t_{i})\left( \sum_{j} \beta_{j} \right)(y_{i}-\alpha-{\log\left( \mathbf{c}_{\mathbf{i}} \right)}^{\text{T}}\boldsymbol{\beta}) \right]$$

(S5)

Collecting all terms in log(*t_i_*)^2^ and log(*t_i_*) from both the likelihood and the prior in t and completing the square gives:

$\sum_{i=0} a_{i}\left( \log\left( t_{i} \right)-\frac{2b_{i}}{a_{i}\log(t_{i})} \right)^{2}-\sum_{i=0} \frac{b_{i}^{2}}{a_{i}}+\left\| \mathbf{y}-\alpha-log(\mathbf{C})\boldsymbol{\beta} \right\|^{2}$ (S6)

where $a_{i}=\left( \sum_{j} \beta_{j} \right)/{\sigma^{2}}+\frac{1}{\sigma_{t}^{2}}$; and $b_{i}={\left( \sum_{j} \beta_{j} \right)\left( y_{i}-\alpha-{\log\left( \mathbf{c}_{\mathbf{i}} \right)}^{\text{T}}\boldsymbol{\beta} \right)}/{\sigma^{2}}+\mu_{t}/\sigma_{t}^{2}$

The complete prior and likelihood are then

$$p\left( \mathbf{y}|\sigma,\alpha,\boldsymbol{\beta},\mathbf{t} \right)p\left( \mathbf{t} \right)=$$

$$\frac{1}{\sqrt{2\pi\sigma^{2}}}exp\left[ -\frac{1}{2}\left( -\sum_{i} \frac{b_{i}^{2}}{a_{i}}+\left\| \mathbf{y}-\alpha-log(\mathbf{C})\boldsymbol{\beta} \right\|^{2} \right) \right]\prod_{i} \frac{1}{{(t}_{i}\sqrt{2\pi\sigma_{t}^{2})}}exp\left[ -\frac{1}{2a_{i}{(\log\left( t_{i} \right)-2b_{i}/a_{i})}^{2}} \right]$$

(S7)

Marginalizing over every *t_i_* we get:

$$\int_{0}^{\infty} p\left( y | \sigma,\alpha,\boldsymbol{\beta},t \right)p\left( t \right)dt$$

$$=\frac{1}{\sqrt{2\pi\sigma^{2}}}exp\left[ -\frac{1}{2}\left( -b^{2}/a+\left\| \mathbf{y}-\alpha-log(\mathbf{C})\boldsymbol{\beta} \right\|^{2} \right) \right]\int_{0}^{\infty} \frac{1}{{(t}_{i}\sqrt{2\pi\sigma_{t}^{2})}}\exp\left[ {-1/2a(\log\left( t \right)-\frac{2b}{a}log(t)}^{2} \right]dt$$

$=\frac{1}{\sqrt{2\pi\sigma^{2}}}exp\left[ -\frac{1}{2}\left( -\sum_{i} b_{i}^{2}/a_{i}+\left\| \mathbf{y}-\alpha-log(\mathbf{C})\boldsymbol{\beta} \right\|^{2} \right) \right]\prod_{i} \sqrt{2\pi/a_{i}}$ (S8)

The marginalized posterior is then:

$p\left( \sigma,\alpha,\boldsymbol{\beta} | \mathbf{y} \right)=\frac{1}{\sqrt{2\pi\sigma^{2}}}\exp\left[ -\frac{1}{2}\left( -\sum_{i} \frac{b_{i}^{2}}{a_{i}}+\left\| \mathbf{y}-\alpha-\log\left( \mathbf{C} \right)\boldsymbol{\beta} \right\|^{2} \right) \right]\frac{p\left( \alpha\right)p\left( \boldsymbol{\beta} \right)p(\sigma^{2})}{Z\times\sqrt[n]{2\pi/a}}$ (S9)

Where Z is the (irrelevant) normalizing constant

**Table A**: Diet compositions for the animal phase

|  |  | **High Fat diets** | | | | |
| --- | --- | --- | --- | --- | --- | --- |
| **Ingredient** | **Control** | **HF-Control** | **HF-WB** | **HF-OB** | **HF-RS** | **HF-FOS** |
| Casein | 200.0 | 190.0 | 169.2 | 152.7 | 190.0 | 190.0 |
| L-Cystine | 3.0 | 3.0 | 3.0 | 3.0 | 3.0 | 3.0 |
| Corn Starch | 387.5 | 173.3 | 158.4 | 110.3 | 155.3 | 172.6 |
| Maltodextrin 10 | 122.0 | 68.3 | 53.4 | 5.3 | 50.3 | 67.6 |
| Sucrose | 100.0 | 283.3 | 268.4 | 220.3 | 265.3 | 282.6 |
| Cellulose, BW200 | 70.0 | 70.0 | 3.3 | 40.0 | 40.0 | 40.0 |
| High Amylose Corn Starch | 0.0 | 0.0 | 0.0 | 0.0 | 83.9 | 0.0 |
| Oligofructose | 0.0 | 0.0 | 0.0 | 0.0 | 0.0 | 32.2 |
| Wheat Bran | 0.0 | 0.0 | 133.3 | 0.0 | 0.0 | 0.0 |
| Oat Bran | 0.0 | 0.0 | 0.0 | 260.9 | 0.0 | 0.0 |
| Butter fat, anhydrous | 0.0 | 44.2 | 44.2 | 44.2 | 44.2 | 44.2 |
| Soybean Oil | 70.0 | 120.3 | 120.3 | 120.3 | 120.3 | 120.3 |
| t-Butylhydroquinone | 0.014 | 0.014 | 0.014 | 0.014 | 0.014 | 0.014 |
| Mineral Mix | 35 | 35 | 35 | 35 | 35 | 35 |
| Vitamin Mix | 10 | 10 | 10 | 10 | 10 | 10 |
| Choline Bitartrate | 2.5 | 2.5 | 2.5 | 2.5 | 2.5 | 2.5 |
| Total | 1000.01 | 1000 | 1000.99 | 1004.51 | 1000.00 | 1000.039 |

Diet abbreviations: High fat control: HF-Control; high fat wheat bran: HF-WB; high fat oat bran: HF-OB; high fat resistant starch: HF-RS; high fat fructooliogsaccharides: HF-FOS.

**Table B**: Bacteria associated with fecal butyrate excretion in rats.

| **Taxon** | $\hat{\boldsymbol{p}}$ | ${\hat{\boldsymbol{\beta}}}_{\boldsymbol{included}}$ | **% of Total** | **Family** | **Genus** |
| --- | --- | --- | --- | --- | --- |
| Otu00098 | 0.673 | 0.025 | 0.1090 | Bacteria_unclassified | Bacteria_unclassified |
| Otu00187 | 0.600 | 0.020 | 0.0384 | Ruminococcaceae | Ruminococcaceae_unclassified |
| Otu00313 | 0.591 | 0.019 | 0.0100 | Ruminococcaceae | Ruminococcaceae_unclassified |
| Otu00399 | 0.581 | 0.019 | 0.0059 | Lachnospiraceae | Lachnospiraceae_unclassified |
| Otu00031 | 0.577 | 0.019 | 0.5586 | Lachnospiraceae | Lachnospiraceae_unclassified |
| Otu00326 | 0.558 | 0.018 | 0.0083 | Lachnospiraceae | uncultured |
| Otu00216 | 0.551 | -0.017 | 0.0285 | Ruminococcaceae | Incertae_Sedis |
| Otu00085 | 0.532 | 0.017 | 0.1328 | Lachnospiraceae | Lachnospiraceae_unclassified |
| Otu00057 | 0.513 | 0.015 | 0.2465 | Lachnospiraceae | Lachnospiraceae_unclassified |
| Otu00069 | 0.499 | -0.014 | 0.1669 | Lachnospiraceae | Lachnospiraceae_unclassified |
| Otu00262 | 0.475 | 0.013 | 0.0177 | Lachnospiraceae | Incertae_Sedis |
| Otu00182 | 0.434 | 0.010 | 0.0390 | Lachnospiraceae | Lachnospiraceae_unclassified |
| Otu00482 | 0.422 | -0.011 | 0.0012 | Ruminococcaceae | Ruminococcaceae_unclassified |
| Otu00170 | 0.398 | 0.009 | 0.0460 | Lachnospiraceae | Lachnospiraceae_unclassified |
| Otu00108 | 0.395 | 0.011 | 0.0935 | Clostridiales_unclassified | Clostridiales_unclassified |
| Otu00547 | 0.395 | -0.008 | 0.0004 | Ruminococcaceae | Incertae_Sedis |
| Otu00324 | 0.392 | 0.008 | 0.0085 | Lachnospiraceae | Lachnospiraceae_unclassified |
| Otu00367 | 0.384 | -0.009 | 0.0098 | Ruminococcaceae | Ruminococcus |
| Otu00167 | 0.383 | 0.008 | 0.0473 | Lachnospiraceae | Lachnospiraceae_unclassified |
| Otu00111 | 0.382 | 0.010 | 0.0880 | Lachnospiraceae | Incertae_Sedis |
| Otu00219 | 0.355 | 0.007 | 0.0276 | Lachnospiraceae | Incertae_Sedis |
| Otu00227 | 0.350 | -0.007 | 0.0244 | Erysipelotrichaceae | Incertae_Sedis |
| Otu00203 | 0.349 | 0.006 | 0.0331 | Lachnospiraceae | Lachnospiraceae_unclassified |
| Otu00058 | 0.343 | -0.007 | 0.2279 | Lachnospiraceae | Blautia |
| Otu00146 | 0.340 | -0.007 | 0.0607 | Lachnospiraceae | Lachnospiraceae_unclassified |
| Otu00320 | 0.337 | -0.006 | 0.0091 | Ruminococcaceae | Incertae_Sedis |
| Otu00063 | 0.336 | 0.008 | 0.1947 | Lachnospiraceae | Blautia |
| Otu00692 | 0.334 | 0.001 | 0.0001 | Firmicutes_unclassified | Firmicutes_unclassified |
| Otu00112 | 0.332 | 0.006 | 0.0874 | Ruminococcaceae | Ruminococcus |
| Otu00124 | 0.332 | -0.006 | 0.0752 | Ruminococcaceae | Incertae_Sedis |
| Otu00134 | 0.332 | 0.008 | 0.0688 | Lachnospiraceae | Lachnospiraceae_unclassified |
| Otu00181 | 0.330 | -0.007 | 0.0397 | Ruminococcaceae | Incertae_Sedis |
| Otu00093 | 0.329 | 0.007 | 0.1132 | Clostridiales_unclassified | Clostridiales_unclassified |
| Otu00422 | 0.327 | 0.005 | 0.0040 | Lachnospiraceae | uncultured |
| Otu00239 | 0.319 | 0.006 | 0.0216 | Lachnospiraceae | Lachnospiraceae_unclassified |
| Otu00331 | 0.318 | 0.006 | 0.0078 | Lachnospiraceae | Lachnospiraceae_unclassified |
| Otu00251 | 0.317 | 0.006 | 0.0200 | Lachnospiraceae | uncultured |
| Otu00356 | 0.317 | 0.005 | 0.0065 | Ruminococcaceae | Ruminococcaceae_unclassified |
| Otu00678 | 0.314 | -0.001 | 0.0001 | Prevotellaceae | Prevotella |
| Otu00229 | 0.313 | -0.007 | 0.0242 | Lachnospiraceae | Incertae_Sedis |
| Otu00122 | 0.312 | -0.005 | 0.0761 | Lachnospiraceae | Lachnospiraceae_unclassified |
| Otu00153 | 0.311 | -0.007 | 0.0573 | Enterococcaceae | Enterococcus |
| Otu00211 | 0.309 | 0.005 | 0.0351 | Firmicutes_unclassified | Firmicutes_unclassified |
| Otu00115 | 0.308 | -0.006 | 0.0824 | Lachnospiraceae | Incertae_Sedis |
| Otu00612 | 0.308 | 0.001 | 0.0001 | Aerococcaceae | Flacklamia |
| Otu00206 | 0.303 | 0.006 | 0.0320 | Ruminococcaceae | Ruminococcaceae_unclassified |
| Otu00415 | 0.301 | 0.005 | 0.0038 | Firmicutes_unclassified | Firmicutes_unclassified |

**Fig A.** Characteristics of original rat community data and data simulated following the procedure outlined in the text. Number of detected OTUs in each sample (Number of non-zero OTUs). The average abundance of each OTU is determined from ~100 samples (or simulations).

**Fig B.** BRACoD results for acetate (top), propionate (middle) and isobutryate (bottom). The inclusion probability $\hat{p}$ is plotted as a function of the regression coefficient $\hat{\beta}_{included}$ determined from BRACoD analysis of rat experimental data. Dotted horizontal line shows cut point = 0.3. Contributors are represented as filled circles (black) while non-contributing OTUs are represented as open circles (white) using a cut point value of 0.3.

**Fig C.** ROC curves showing model performance on a single simulated dataset with 20 contributing bacteria and 119 samples. For BRACoD and clr-SS, the inclusion probability $\hat{p}$ was varied to obtain different TPR and FPR values. For clr-LASSO, the regularization strength (lambda) was varied. The points on the curve correspond to the cut points used to determine TPR and FPR. For BRACoD and clr-SS this was an inclusion probability ($\hat{p}$) > 0.3 and for clr-LASSO this was the best lambda identified by cross validation. Different versions of the simulated data produce different ROC curves (see Figure 4, which demonstrates performance metric variation across simulations). This figure was, therefore, generated using 100 simulations. The areas under the curve are: 0.9114 (BRACoD), 0.8798 (clr-LASSO) and 0.8162 (clr-SS).


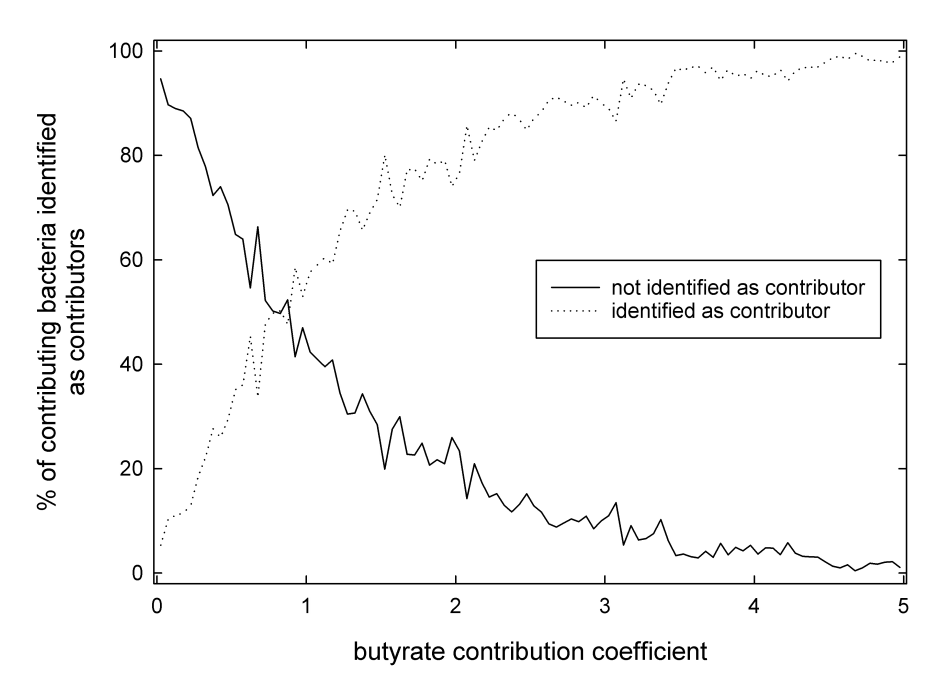


**Fig D.** BRACoD preferentially identifies bacteria with a large contribution coefficient. Relationship between the contribution coefficient and the percentage of contributing bacteria that were identified as contributors (dotted line). The percentage of contributing bacteria that were not identified as contributors is also shown (solid line).

**Fig E.** Method parameters as a function of inclusion cut point ($\hat{p}$) for BRACoD (top) and clr-SS (bottom) algorithms. BRACoD TPR, FPR, precision, and accuracy parameters (top) are from Figure 3A and are included for comparison.

**Fig F.** BRACoD analysis of the association between bacterial genera (top) and bacterial family (bottom). The inclusion probability $\hat{p}$ is plotted as a function of the regression coefficient $\hat{\beta}_{included}$ determined from BRACoD analysis of rat experimental data. Dashed horizontal line shows cut point = 0.3. Contributors are represented as filled circles (black) while non-contributing OTUs are represented as open circles (white) using the cut point value of 0.3.
